# The Dominant Role of Dietary Differences in Shaping the Intestinal Microbial Communities of Grass Carp, Carp, and Crucian Carp in a Saline–Alkali Lake in Xinjiang During Winter

**DOI:** 10.3390/microorganisms13112572

**Published:** 2025-11-11

**Authors:** Ruomei Ma, Yaya Chen, Xiande Chen, Jiaqi Zhang, Changcai Liu, Liting Yang, Yong Song, Zhen Sun, Xuyuan Lin, Tao Ai, Daoquan Ren, Sheng’ao Chen

**Affiliations:** 1 State Key Laboratory Incubation Base for Conservation and Utilization of Bio-Resource in Tarim Basin, Tarim Research Center of Rare Fishes, College of Life Sciences and Technology, Tarim University, Alar 843300, China; 10757231129@stumail.taru.edu.cn (R.M.); m19882959196@163.com (Y.C.); cxd2113@163.com (X.C.); 10757242168@stumail.taru.edu.cn (J.Z.); 18710677465@163.com (C.L.); 10757231127@stumail.taru.edu.cn (L.Y.); songyongdky@126.com (Y.S.); 2 East China Sea Fisheries Research Institute, Chinese Academy of Fishery Sciences, Shanghai 200090, China; sunzhen@ecsf.ac.cn; 3 Fishery Technical Extension Station, Xinjiang Production Construction Group, Urumqi 830000, China; btsc229@sina.com (X.L.); taoai202506@163.com (T.A.)

**Keywords:** intestinal microbiota, 16S rRNA, fooding habit, microbial ecology, saline–alkali lakes of Xinjiang

## Abstract

In this study, gut microorganisms of herbivorous grass carp, omnivorous carp, crucian carp, and aquatic microorganisms were collected from natural salt–alkali lakes and ponds in Xinjiang in winter to analyze their community structures. We sequenced 16S rRNA amplicons to investigate the composition and function of the microorganisms in the gut. PCoA analysis revealed that the gut microbiota of herbivorous and omnivorous fish formed two distinct clusters. *Proteobacteria*, *Actinobacteria*, *Desulfobacterota*, *Firmicutes*, and *Chloroflexia* are the dominant bacteria in the gut of fish. Proteobacteria, Bacteroidetes, Actinobacteria, Cyanobacteria, and Gram-negative bacteria are the dominant bacteria in the water. Carbohydrate- and protein-degrading bacteria, such as *Desulfofustis*, *Lactiplantibacillus*, and *Vibrio*, were predominant in omnivorous fish (CC and GRC), while cold-resistant bacteria, such as *Shewanella* and *Psychromonas*, were colonized in grass carp. This suggests that the same environment does not lead to similar gut bacteria, and that specific endogenous factors play a far more important role in shaping the microbiota composition than environmental factors.

## 1. Introduction

The gastrointestinal tract of animals consists of a very complex and dynamic microbial ecosystem [1]. Within the microbial population, bacteria are the main colonizers of the gastrointestinal tract. The gut microbiota of fish plays an important role in nutrition [2], nutritional metabolism, immunity [3], and adaptation to the environment [4]. The presence of the gut microbiota creates an excellent microecological environment for the host, and the symbiotic properties of fish provide suitable conditions for the proliferation of the gut microbiota. Using zebrafish and rainbow trout (*Oncorhynchus mykiss*) and other important fish models [5], the mechanism of interaction between fish host and microbiota has been investigated, including the regulation of microbiota on fish nutrition, immunity, and other physiological functions, and the mutual regulation of host immunological components and non-immune factors on the microbiota [6]. In this process, a dynamic relationship between gut flora and host has evolved, characterized by interdependencies and constraints [1,7]. Diet is considered an important factor influencing the gut microbiota. Therefore, diet has been reported to be the main cause of changes in the composition of the microbiota of rainbow trout [8,9]. Destruction of the intestinal microbiota due to dietary habits usually affects the function of the digestive organ by impairing the production of bacterial digestive enzymes [10]. However, environmental factors also have an important influence on the gut microbial community of fish, with water and sediment being the most important environmental factors [4]. It is worth noting that there are species-specific differences in the intensity of the effects of water and sediment on the gut microbiota of fish [11]. Li et al. [12] investigated the gut microflora of different carp species (*Ctenopharyngodon idellus*, *Carassius cuvieri*, and *Hypophthalmichthys nobilis*) reared in the same pond. From the survey results, the gut microbial communities of the different carp species were dominated by *Fusobacteria*, *Firmicutes*, *Proteobacteria*, and *Bacteroidetes*, but the abundance of the different species was significantly different, suggesting that the gut flora was caused by species-specific selection pressure.

Grass carp is one of the most important freshwater aquaculture species in China, with the highest aquaculture output among freshwater fishes—its output reached 5.7 million tons in 2015 [13]. Grass carp is known for converting plant biomass into animal protein, relying on its intestinal microbiota to assist in digesting and absorbing plant materials. It mainly feeds on aquatic plants, including higher aquatic plants and underwater terrestrial vegetation. Studies have shown that the intestinal microbial community of grass carp is rich in cellulose-decomposing bacteria, including *Leuconostoc*, *Clostridium*, *Aeromonas*, *Bacteroides*, *Shewanella*, *Cetobacterium*, and *Actinomycetes-related* genera [14,15]. This microbial composition feature has a significant adaptive association with the dietary structure of herbivorous fish dominated by cellulose-rich plant foods [16]. Crucian carp (scientific name: *Carassius auratus*) is another widely popular freshwater farmed fish in China, characterized by strong environmental adaptability (tolerating low oxygen levels, pH fluctuations, and salinity changes) and a fast growth rate [17,18]. As an omnivorous animal, crucian carp exhibits a wide dietary range, including zooplankton, benthic insect larvae, macrophytes, and detrital materials [19]. Such a dietary structure exerts targeted shaping on the gut microbial community through the digestive requirements of food components. Different types of food require bacteria with specific functions to participate in decomposition (e.g., animal-based foods require protease-related bacteria, and plant-based foods require cellulose-degrading bacteria), thereby influencing the composition and abundance of dominant bacterial genera in the gut [20]. A comparative study of three polyculture freshwater fishes (*grass carp*, *crucian carp*, and *bighead carp*, *Hypophthalmichthys nobilis*) in typical freshwater aquaculture lakes in eastern China revealed a significant feature of the gut microbiota of crucian carp: the abundance of *Cetobacterium* in the gut was 2.3–3.1 times higher than that of grass carp and bighead carp [12]. Carp is a common freshwater omnivorous fish, but its diet is meatier, mainly feeding on benthic organisms. In the lake ecosystem, the composition of the intestinal microbial community of carp is closely associated with its omnivorous feeding habit (with a preference for animal-based food) [21]. Studies have shown that the intestinal microorganisms of carp mainly include *Clostridium*, *Cetobacterium*, and *Halomonas*, and there are also bacteria that can produce proteases, such as *Halomonas* and *Cetobacterium* [22,23].

Although a large number of studies have been carried out on the gut microbiota of freshwater fish at home and abroad [22], and the regulatory effects of feeding habits and environmental factors on the structure of the microbiota have been clarified, these studies mostly focus on ordinary freshwater waters such as rivers, lakes, and aquaculture ponds. The conclusions are difficult to directly adapt to the special habitats of high salinity and high alkalinity [24,25]. The community characteristics and functional mechanisms of fish gut microbiota in saline–alkali lakes, especially the interaction between ‘fish gut microbiota-water microbes’, are still lacking systematic discussion, forming a clear research gap. Grass carp, carp, and crucian carp are the most common dominant economic fish in saline–alkali lakes and supporting ponds in Xinjiang. Their intestinal microbial communities are subject to multiple regulations of host physiological characteristics, feeding habits, and living environment (salinity, water temperature). Under the special stress of high salt and high alkali, the intestinal microorganisms of these fish are more likely to help the host adapt to the extreme environment by synthesizing salt-tolerant metabolites and regulating intestinal osmotic pressure balance [26].

There are many natural saline–alkali lakes and ponds distributed in Xinjiang. These unique ecosystems are different from ordinary freshwater waters due to special physical and chemical properties, such as high salinity and alkalinity, forming a unique microbial community structure. At present, there are few joint studies on the intestinal microorganisms and water microorganisms of grass carp, carp, and crucian carp in Xinjiang natural saline–alkali lakes. In this study, by measuring the physical and chemical parameters (salinity, alkalinity, water temperature, dissolved oxygen) of saline–alkali lake water in winter and the 16S rRNA gene sequence of gut–water microorganisms of three fish species, it can be determined whether ‘low temperature will strengthen the screening effect of saline–alkali on microbial community’ and can locate the key functional flora of ‘saline-alkali tolerance + low temperature tolerance’. In-depth exploration of the characteristics of these microbial communities will help to reveal the relationship between fish and environmental microorganisms, as well as the function and mechanism of microorganisms in the saline–alkali lake ecosystem, aiming to provide a scientific basis for the protection and sustainable utilization of the saline–alkali lake aquatic ecosystem.

## 2. Materials and Methods

### 2.1. Sample Collection

**Figure 1:**
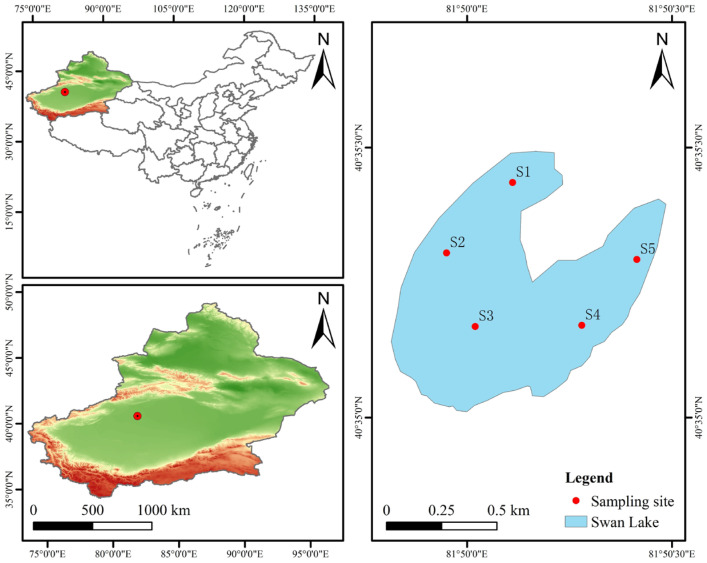
The geographical location of the saline–alkaline lake and lake water sampling points (S1–S5).

In this study, a high saline–alkali lake pool (hereinafter referred to as saline–alkali lake) in Alar City, Xinjiang Uygur Autonomous Region, was selected as the research area. The geographical coordinates were 81°50′30′ E and 40°35′30′ N (Figure 1). The lake is named Swan Lake, which is naturally formed by the drainage of the alkaline drainage canal in the Tarim River irrigation area, with a total area of 0.34 square kilometers. In February 2025, sticky nets were used for fishing in the sampling waters. We collected samples of three wild fish species, grass carp (*Ctenopharyngodon idellus*), crucian carp (*Carassius auratus*), and carp (*Cyprinus carpio*), named the GC group, GRC group, and CC group, respectively. Six individuals were collected for each species, with an average body mass of 1.65 ± 0.5 kg per fish, and a total of eighteen fish were collected. Each fish was euthanized with MS-222 (0.6–1.0 g/L) after capture, and the fish was disinfected with 75% anhydrous ethanol. The intestine was dissected from the anus along the abdomen with sterile scissors, and the intestine was dissected from the abdomen, extending from the anus to the region near the tail. Then, it was washed with sterile PBS three times, and the intestinal contents were squeezed out with sterile tweezers. The intestinal contents of every 2 fish were pooled and placed in a 2 mL sterile centrifuge tube to form a sample. Three biological replicates were prepared, and each replicate was analyzed in triplicate. In addition, this study used a portable multi-parameter water quality analyzer (YSI Pro Professional Plus, Yellow Springs, OH, USA) to measure physical and chemical indicators of water bodies such as water temperature (WT, °C), dissolved oxygen (DO, mg/L), pH, salinity (Salinity, ppt), and total dissolved solids (TDS, mg/L) at a depth of 0.5 m on-site. The measurements were taken sequentially at each sampling point, and the results are shown in Table 1.

All the collected samples were immediately frozen in liquid nitrogen until DNA extraction was performed in the laboratory. At the same time, 4 L of surface and bottom water samples were collected at the same location. The surface and bottom water samples were mixed and placed in a sterile water collection bag and then immediately filtered with a 0.22 μm pore size Millipore filter membrane (Millipore, Darmstadt City, Germany). Each filter membrane was used to filter 1 L of the mixed water, and 4 filters (corresponding to 4 L of total water) were collected. The samples were quickly frozen in liquid nitrogen and transported to the laboratory, where they were stored at −80 °C. Finally, the samples and dry ice were sent to Shanghai Biotechnology Co., Ltd. (Shanghai, China) for high-throughput sequencing analysis of the amplicons.

### 2.2. DNA Extraction, PCR Amplification, and 16S rRNA Sequencing

Total genomic DNA samples were extracted using the Mag Beads Fast DNA Kit for Soil (116564384) (MP Biomedicals, Irvine, CA, USA), following the manufacturer’s instructions, and stored at −20 °C prior to further analysis. The quantity and quality of extracted DNAs were measured using a NanoDrop NC2000 spectrophotometer (Thermo Fisher Scientific, Waltham, MA, USA) and agarose gel electrophoresis, respectively.

PCR amplification of the bacterial 16S rRNA genes V3–V4 region was performed using the forward primer 338F (5′-ACTCCTACGGGAGGCAGCA-3′) and the reverse primer 806R (5′-GGACTACHVGGGTWTCTAAT-3′). Sample-specific 7 bp barcodes were incorporated into the primers for multiplex sequencing. The PCR components contained 5 μL of buffer (5×), 0.25 μL of Fast PFU DNA Polymerase (5 U/μL), 2 μL (2.5 mM) of dNTPs, 1 μL (10 μM) of each forward and reverse primer, 1 μL of DNA template, and 14.75 μL of ddH_2_O.

The PCR amplification products were purified using Vazyme VAHTSTM DNA purification magnetic beads (Nanjing Novartis Biotechnology Co., Ltd., Nanjing, China) and quantified using a Quant-iT PicoGreen double-stranded DNA detection kit (Invitrogen, Calsbad, CA, USA). After the completion of the individual quantitative steps, the amplification products were mixed at equal concentrations, and the 2 × 250 bp double-ended sequencing was performed by Shanghai Pasenuo Biotechnology Co., Ltd. (Shanghai, China) using the Illumina NovaSeq platform with the NovaSeq 6000 SP reagent kit (Illumina, San Diego, CA, USA) (500 cycles).

### 2.3. Sequencing Data Analysis

Microbiome bioinformatics analysis was performed using QIIME2 2024.5 [27]. Briefly, raw sequence data were demultiplexed using the demux plugin, followed by primer trimming using the cutadapt plugin [28]. Sequences were then quality filtered, denoised, and merged, and the chimera was removed using the DADA2 plugin [29]. Non-singleton amplicon sequence variants (ASVs) were aligned with mafft [30] and used to construct a phylogeny with fasttree2 [31]. The number of sequences per sample was recorded, and taxonomy was assigned to ASVs using the classify-sklearn naïve Bayes taxonomy classifier in featureclassifier plugin [32] against the SILVA Release 138 Database [33].

### 2.4. Bioinformatics and Statistical Analysis

The diversity plug-in was used to calculate the α diversity index (Chao1, Shannon, Simpson, ACE) to comprehensively evaluate the richness and evenness of the microbial community. The non-parametric Kruskal–Wallis test was used to detect the significant differences in the α diversity index of the four groups. *p* < 0.05 was considered statistically significant. The Bray–Curtis index, non-metric multidimensional scaling analysis (NMDS), and unweighted pair-group average method based on arithmetic mean (UPGMA) hierarchical clustering [34] were used to explore the structural differences of microbial communities among samples, and the R script (version 4.3.3) ggtree package (version 3.12.0) was used for visualization. QIIME2 (version 2024.5) was used to evaluate the significance of differences in microbial community structure between groups by permutation similarity analysis (ANOSIM) [35,36]. The Venn diagram is generated using the R package (version 4.3.3) VennDiagram, based on the occurrence of ASV in the sample to show the common and unique ASV [37] between the sample or group. The linear discriminant analysis (LDA) effect size (LEfSe) method was used to test the differential species [38] in different samples. The alpha analysis parameter was set to 0.05 based on the genus level, and the threshold was set to LDA = 4. PICRUSt2 (Phylogenetic Investigation of Communities by Reconstruction of Unobserved States) [39] was used to predict microbial function based on the MetaCyc database (https://metacyc.org/) (accessed on 29 April 2025) and KEGG database (https://www.kegg.jp/) (accessed on 29 April 2025). Based on the Wilcoxon rank sum test, the significant difference in functional abundance between species was tested. The results were plotted using the Tutools platform (http://cloudtutu.com.cn/) (accessed on 29 April 2025). Finally, Spearman rank correlation was used to study the relationship between environmental factors and microbial species abundance, and the pairwise correlation and its statistical significance were obtained.

## 3. Results

### 3.1. Diversity Analysis of Microbial Community

**Figure 2:**
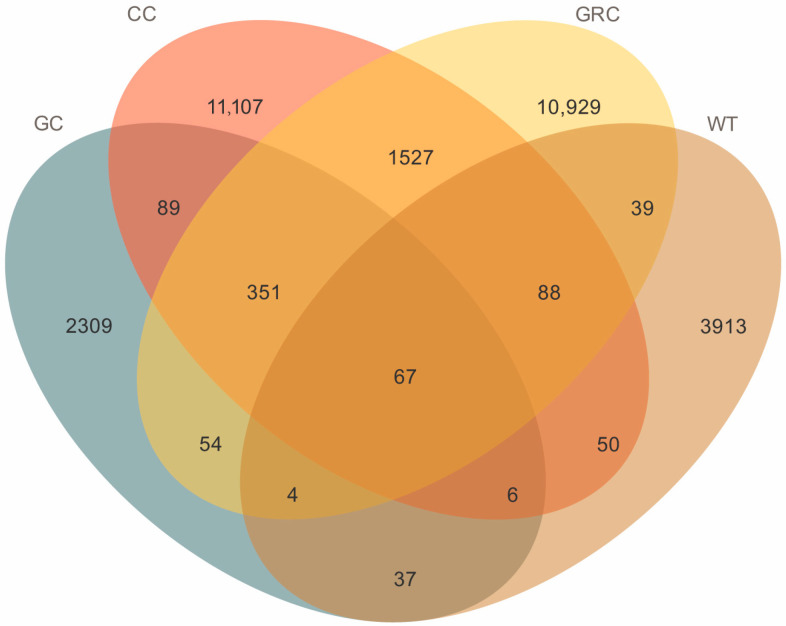
ASV Venn analysis diagram. GC: grass carp; CC: carp; GRC: crucian carp; WT: water samples.

A total of 2,389,953 high-quality sequences were obtained, with an average of 77,095 sequences per sample. The total number of ASVs was 29,881, including 72 phyla, 175 classes, 440 orders, 874 families, 1750 genera, and 2415 species. It can be seen from the Venn diagram that there were 67 common genera across the four groups (GC, CC, GRC, WT). The CC (*carp*) and GRC (*crucian carp*) groups had the highest number of genera (10,929 and 11,107, respectively), while grass carp and water samples had fewer genera, 2309 and 3913, respectively. The results show that there are obvious differences in bacterial communities between fish intestines and between fish intestines and water. In winter, the intestinal flora of grass carp is much lower than that of carp and crucian carp, and the number of microorganisms in the water body is quite different from that of fish intestines, indicating that the water body has little effect on the intestinal tract in winter (Figure 2).

**Figure 3:**
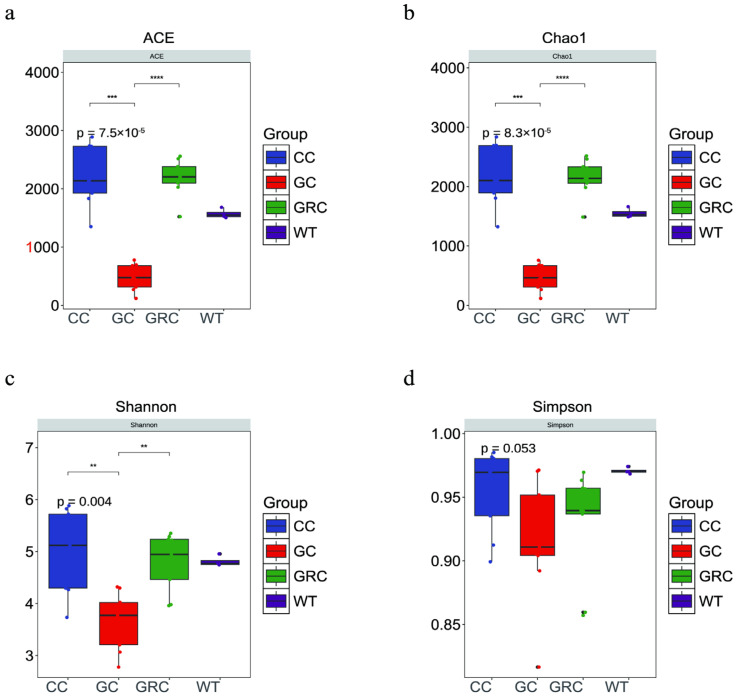
Box diagram of microbial alpha diversity of grass carp, carp, crucian carp, and water samples. GC: grass carp; CC: carp; GRC: crucian carp; WT: water sample. (**a**) ACE index; (**b**) Chao1 index; (**c**) Shannon index; (**d**) Simpson index; (**) *p* < 0.01 and (***) *p* < 0.001, (****) *p* < 0.0001.

The microbial diversity and richness of the samples were compared by the ACE index, Chao1 index, Shannon index, and Simpson index. In these four groups, the intestinal microflora of carp showed the highest value in all four alpha diversity indexes (Table 2). The results showed that the intestinal microbial diversity of grass carp was significantly lower than that of carp and crucian carp (*p* < 0.01), and the microbial richness of grass carp was significantly lower than that of carp and crucian carp (*p* < 0.001) (Figure 3).

**Figure 4:**
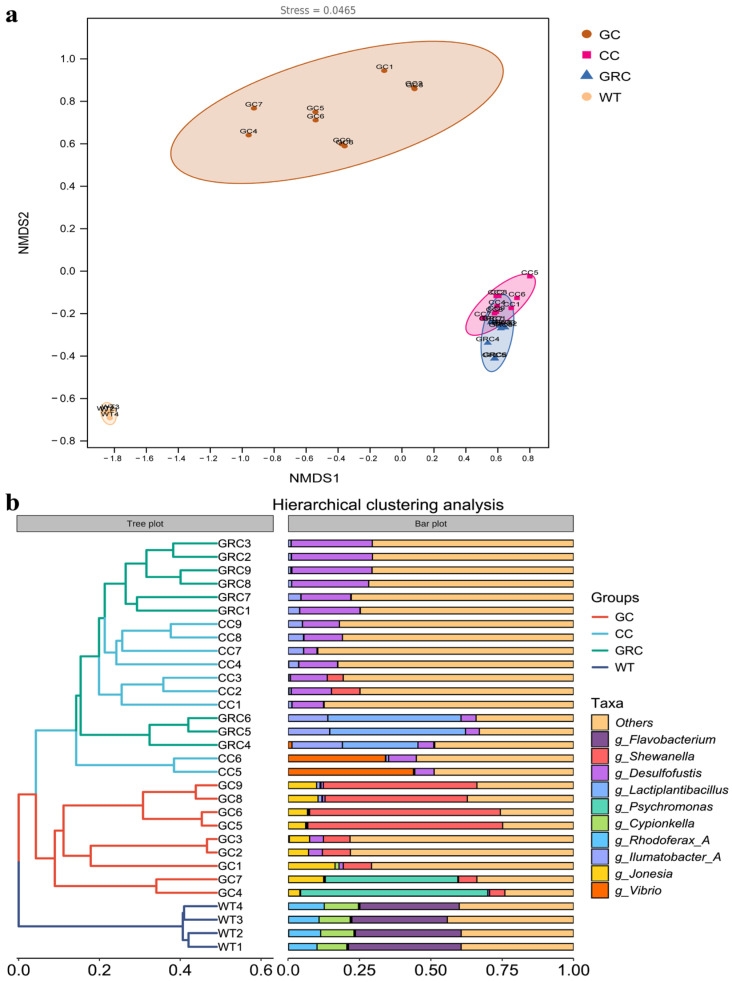
NMDS (**a**) based on Bray–Curtis distance and hierarchical clustering analysis (**b**). GC: grass carp; CC: carp; GRC: crucian carp; WT: water samples.

Beta diversity analysis was performed on the intestinal contents and water microorganisms of the three fish species to explore the similarities and differences in microbial composition. As shown in Figure 4a, the results of NMDS clearly showed that there were significant differences between the intestinal microorganisms of the three fish species and the water microorganisms, as well as between omnivorous fish and herbivorous fish. In the intestinal samples of fish, there was overlap between carp and crucian carp samples, indicating that there was little difference between the two colonies, while there were significant differences between carp and crucian carp samples and grass carp and water samples, respectively. On the whole, it can be divided into three branches, among which GC samples are clustered into one branch, WT samples are clustered into one branch, and individual samples between GRC and CC samples are clustered into one branch (Figure 4b). These results indicate that the composition of the intestinal flora of carp and crucian carp has higher similarity.

### 3.2. Microbial Community Composition

**Figure 5:**
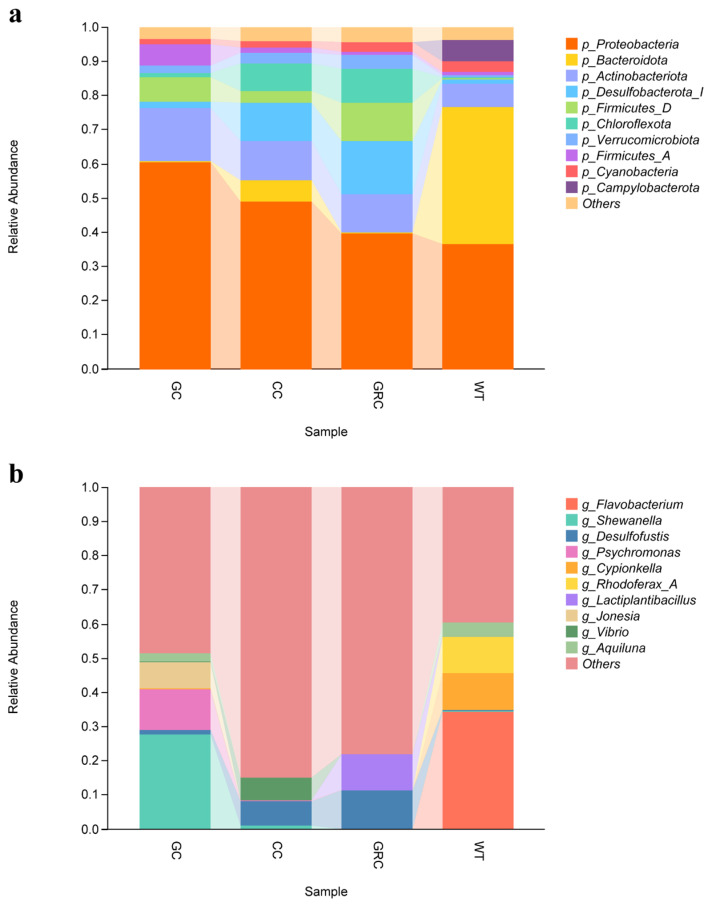
The abundance composition of gut microbiota at the phylum level (**a**) and the genus level (**b**). GC: grass carp; CC: carp; GRC: crucian carp; WT: water samples.

At the phylum level, Proteobacteria was the most abundant phylum in the intestines of grass carp, carp, and crucian carp, accounting for 60.6%, 49.1%, and 39.7%, respectively. This indicates extensive colonization characteristics in the intestinal tract of aquatic organisms. The abundance of *Actinobacteria* in the intestines of grass carp was significantly higher than that of other fish, accounting for 15.4%; the abundance of *Cyprinus carpio* (11.4%) and *Carassius auratus* (11.2%) was similar. The proportion of *Bacteroidetes* in water samples was 40.1%, which was the absolute dominant phylum, while the abundance in fish intestine was extremely low, which was 0.3% in grass carp and *Carassius auratus* and 6.1% in *Cyprinus carpio*. The abundance of *Desulfobacterota* was higher in the intestines of omnivorous carp (11.3%) and crucian carp (15.5%) (Figure 5a).

At the genus level, the top ten genera of the main bacteria in the intestine and water are shown in Figure 5b: *Flavobacterium*, *Shewanella*, *Desulfofustis*, *Psychromonas*, *Cypionkella*, *Rhodoferax*, *Lactobacillus*, *Jonesia*, *Vibrio*, and *Aquiluna*. The dominant bacteria in grass carp are *Shewanella* and *Psychromonas*. The dominant genera in water are *Flavobacterium* (phylum Bacteroidetes), *Cypionkella*, and *Rhodobacter*. The dominant bacteria in carp and crucian carp are *Desulfofustis*, *Vibrio*, and *Lactobacillus*.

**Figure 6:**
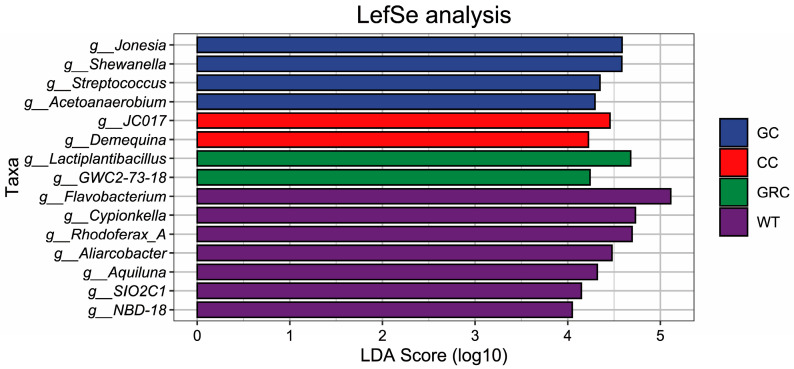
LEfSe analysis of water and fish gut microbiota based on genus level (LDA > 4). GC: grass carp; CC: carp; GRC: crucian carp; WT: water samples.

The results showed that *Jonesia*, *Shewanella*, *Streptococcus*, and *Acetoanaerobium* were identified as the significant difference flora in the grass carp group, while *g_JC017* and *Demequina* were identified as the significant difference flora in the carp group. *g_Lactiplantibacillus* and *g_GWC2-73-18* were identified as the significant difference flora in the crucian carp group; *g_Flavobacterium*, *Cypionkella*. *Rhodoferax*, *Aliarcobacter*, *Aquiluna*, *g_SIO2C1*, and *g_NBD-18* were identified as significantly different bacteria in water. LDA values were greater than 4. In general, the results of LEfSe analysis and LDA discrimination are consistent (Figure 6).

### 3.3. Functional Pathways with Significant Differences Between the Four Groups

**Figure 7:**
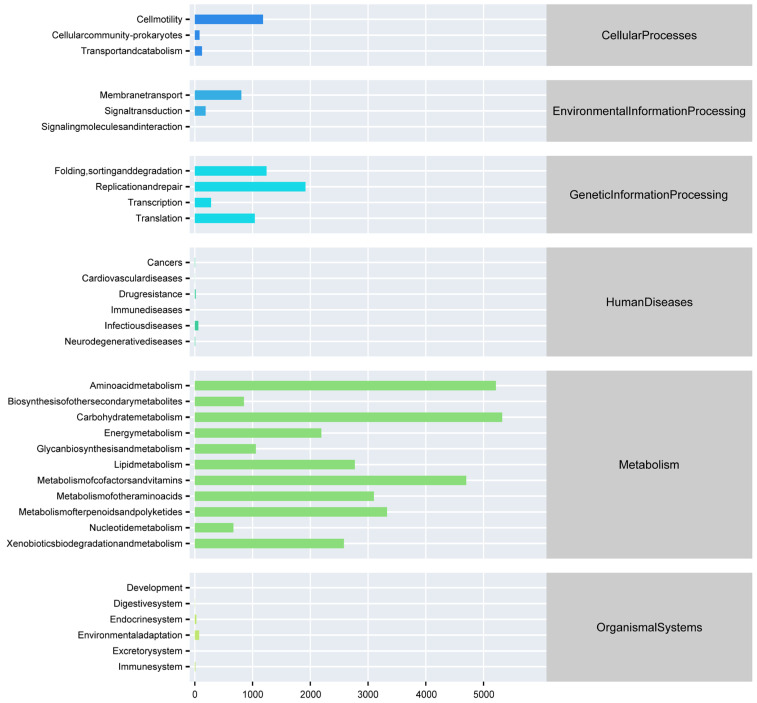
The functional abundance distribution between fish and the water body.

We used PICRUSt to predict the function of 16S rRNA gene amplicons to obtain gene function annotation information at pathway levels 1 and 2 (Figure 7). In the primary pathway, the abundance of metabolism is high, followed by gene information processing and cellular processes. In the secondary pathway, the most abundant functions are amino acid metabolism, carbohydrate metabolism, cofactors, and vitamin metabolism, which are components of metabolism in the primary pathway.

**Figure 8:**
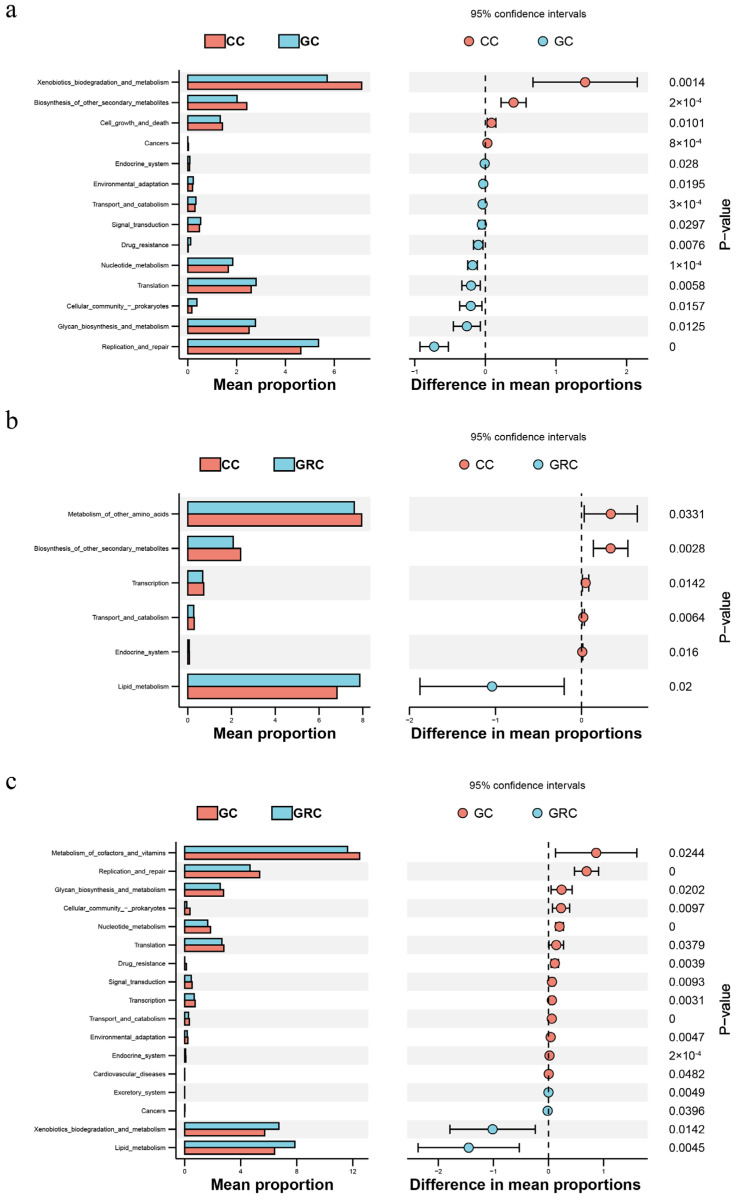
CC and GRC (**a**) based on a Wilcoxon rank sum test; CC and GC (**b**); significant differences in functional abundance between GC and GRC (**c**) (*p* < 0.05). GC: grass carp; CC: carp; GRC: crucian carp.

Figure 8 of the predicted KEGG pathway also shows that there may be a huge difference between the ongoing microbial processes in the gut communities of herbivorous and omnivorous fish. The abundance of cofactors and vitamin metabolism, environmental adaptability, transport and catabolism, signal transduction, transcription, drug resistance, nucleotide metabolism, translation, cell community-prokaryote, glycoside biosynthesis and metabolism, replication and repair, and endocrine system in grass carp were significantly higher than those in common carp and crucian carp (*p* < 0.05). Compared with grass carp, the biodegradation and metabolism of xenobiotics in omnivorous fish and in lipid metabolism are more prominent, followed by cell growth and death.

### 3.4. Correlation Analysis

**Figure 9:**
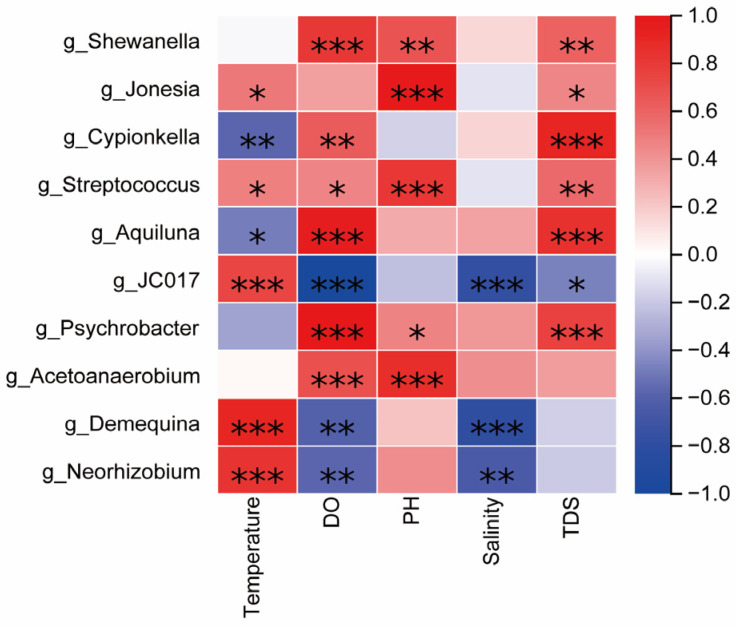
The correlation heatmap between the relative abundance of bacteria and the physical and chemical factors of the water body at the genus level, and the color depth indicates the correlation strength; marker (*) *p* < 0.05, (**) *p* < 0.01 and (***) *p* < 0.001.

In order to further explore the response relationship between environmental factors and the abundance of microbial community composition, the Spearman correlation between the top 10 dominant genera of the relative abundance of microorganisms in grass carp, carp, crucian carp, and water and environmental factors was analyzed, and a heatmap (Figure 9) was drawn. It can be seen from the figure that bacteria are related to a variety of environmental factors. *g_Shewanella* and *g_Psychrobacter* were significantly positively correlated with DO, pH, and TDS (*p* < 0.001). *g_Cypionkella* and *g_Aquiluna* were significantly positively correlated with DO and TDS (*p* < 0.001) and significantly negatively correlated with temperature (*p* < 0.05).

## 4. Discussion

The Swan Lake of the 14th Regiment of the Xinjiang Production and Construction Corps is located on the northwest edge of the Taklimakan Desert, the easternmost end of the Tarim Reclamation Area of the First Division, and the easternmost end of the Tarim Irrigation Area. The formation of this water area originated from the 76 million cubic meters of saline–alkali water discharged by the Fourteen Group Town (the south bank of the Tahe River) every year, which was collected into a river, and finally formed a saline–alkali water area of more than 4300 mu, which is a typical inland arid area saline–alkali lake [40]. On the one hand, its environmental parameters are significantly different from those of ordinary freshwater lakes, with a salinity of 8–18‰ (salinity of ordinary freshwater lakes < 1‰) and a pH value of 8.8–10.2 (pH value of ordinary freshwater lakes 6.5–8.0). Affected by the continental climate in arid areas, the temperature difference between day and night can reach more than 15 °C, and the freezing period in winter is up to 4 months, forming a unique living environment of ‘high salinity + seasonal extreme stress (temperature difference, glacial period)’ [41,42]. On the other hand, grass carp, carp, and crucian carp are the dominant populations in Swan Lake, and their gut microbiota is the result of long-term adaptation to ‘high saline-alkali stress + seasonal extreme environmental interference’, which is significantly different from the gut microbiota of common saline–alkali pond fish and freshwater fish. The study of 16S rRNA high-throughput sequencing of intestinal microorganisms in these three fish species is not only an important extension of the research on fish microorganisms in extreme environments but also can reveal the unique interaction mechanism of ‘host (fish) -intestinal flora-environment (high saline-alkali waters)’ in special ecosystems, which has irreplaceable research value.

Studies have shown that *Proteobacteria*, *Firmicutes*, *Bacteroidetes*, *Actinobacteria*, and *Clostridium* are the main microbial groups in the intestine of fish, which together constitute the core microbial community that regulates the diversity and structure of fish intestinal flora [43,44]. At the phylum level, this study found that *Proteobacteria*, *Actinobacteria*, *Bacteroidetes*, *Firmicutes*, and *Desulfobacter* were the dominant phyla in the intestine and water of grass carp, carp, and crucian carp (three kinds of fish), but their relative abundance in the intestine and water was significantly different. The proportion of *Bacteroidetes* in water was as high as 40%, while the proportion of *Bacteroidetes* in the intestines of grass carp, carp, and crucian carp was only about 1%, forming a significant difference. *Actinobacteria* species often produce *antibacterial* substances and growth-promoting substances [45]. Combined with its distribution characteristics in water, it is suggested that it may be more feasible to screen beneficial strains of this phylum from water; the relative abundance of *Proteobacteria* in the intestine and water was the highest, indicating that it had stronger colonization ability in this environment. From crucian carp to carp to grass carp, the relative abundance of *Proteobacteria* and *Actinobacteria* showed a gradual upward trend (Figure 5a). *Firmicutes* are also typical intestinal colonizing bacteria, which is consistent with most previous studies [46,47]. However, when the above representative bacteria were further refined to the genus level, the differences in flora between different fish hosts were significantly enhanced, and each fish had its own unique dominant genus. The main dominant genus in water is *Flavobacterium*. This genus may be involved in the absorption, degradation, and decomposition of organic matter in the cold aquatic environment, while regulating the biomass of planktonic bacteria, which may be the main reason for its becoming the dominant genus in the water body; in addition, LEfSe analysis also confirmed that *Flavobacterium* is one of the indicator bacteria in water (Figure 6), which is consistent with previous research results [48]. The dominant genera of grass carp (herbivorous) are *Shewanella* and *Psychrobacter*. The feeding activity and immune function of grass carp in winter will decrease significantly. The change of intestinal environment may be more conducive to the colonization of *Shewanella* and *Psychrobacter*, which are resistant to low temperature and high salt. The function of these bacteria may be more important in the survival adaptation of grass carp in winter [16,49,50]. In carp and crucian carp (omnivorous), a variety of bacteria capable of degrading carbohydrates and proteins were detected, such as *Desulfobacter*, *Lactobacillus plantarum*, *Vibrio*, etc., which are also the core bacteria in the intestines of these two omnivorous fish. Among them, some *vibrios* can synthesize B vitamins (such as vitamin B1, B2, folic acid) and vitamin K, and their metabolites (such as short-chain fatty acids, organic acids) can also indirectly maintain intestinal microecological balance [51,52]. Environmental factors are the key drivers that regulate the structure and function of microbial communities [53]. In this study, Spearman correlation analysis showed that temperature and dissolved oxygen (DO) were the core driving factors affecting the dominant genera of microorganisms in the intestine and water of grass carp, carp, and crucian carp. Salinity and total dissolved solids (TDS) act on specific microbial groups through ‘niche differentiation’. This result is consistent with the general understanding of ‘multi-factor synergy in shaping microbial communities’ in freshwater ecosystems [54]. It should be noted that this study only focused on the effects of water quality parameters on the microbiome in a single period of winter. Other environmental variables, such as seasonal changes, may indirectly regulate microbial community structure by changing water temperature, nutrient availability, and ribosomal DNA analysis without functional verification. Therefore, future research needs to include such dynamic variables to more comprehensively analyze the dynamic law of microbial–environmental interactions.

The composition and structure of the intestinal flora of fish are usually limited by both biological factors (such as host species differences) and abiotic factors (such as habitat environment) [11,55]. This also leads to significant differences in microbial communities between fish intestinal samples and water samples. The NMDS results showed that the intestinal bacterial communities of different trophic levels of fish formed different clusters. *Cyprinus carpio*, *Carassius auratus*, and *Ctenopharyngodon idellus* formed obvious clusters in NMDS space (Figure 4a). This result is similar to the study of Sullam et al. [53]. In addition, the results of cluster analysis showed that the bacterial communities of carp and crucian carp were clustered together, while the bacterial communities of grass carp and water were clustered together, which indicated that omnivorous fish could be selectively enriched in one group (Figure 4b). The bacterial communities of omnivorous and herbivorous fish are quite different from those in water. These results indicate that there are specific bacterial populations in different species [22]. Studies have confirmed that host species can also affect the α diversity and β diversity of the fish gut microbiome [22]. In this study, the results of α diversity in winter showed that there were significant differences in microbial richness between intestinal and water samples of common carp, crucian carp, and grass carp (*p* < 0.05) (Table 1). The α diversity index of common carp and crucian carp was the highest, followed by the water body, and grass carp was the lowest, which may be due to the stagnation of aquatic plant growth caused by the decrease in water temperature in winter [56]. In contrast, omnivorous fish have a wider food spectrum and can flexibly eat animal debris (such as zooplankton, benthic invertebrate carcasses), humus (rich in microorganisms and organic debris), and even small algae or plant tender leaves in winter [57]. Therefore, the diversity index of omnivorous fish is higher. This result is consistent with previous research results [11]. In addition, our results show that the gut microbiota has the highest expression level in metabolism-related aspects, which reflects the deep symbiotic relationship between microorganisms and their hosts during long-term co-evolution [58,59]. The KEGG pathway (level 2) is enriched in diet-related functional categories, such as biodegradation and metabolism of omnivorous fish and lipid metabolism, which are different among different species (Figure 8a,c). This may be due to the feeding habits of omnivorous fish driving their nutritional needs. Lipids are more efficient energy storage substances than carbohydrates and proteins, and the living environment of omnivorous fish (such as freshwater lakes and rivers) has large fluctuations in food resources (seasonal changes, competitive pressures) [60]. Therefore, it is necessary to maintain balance by strengthening lipid metabolism. As mentioned above, this is consistent with the fact that the intestinal flora of carp and crucian carp (such as *Proteobacteria* and *Actinobacteria*) directly drives the high activity of the lipid metabolism pathway by highly expressing functional genes related to lipid metabolism to compensate for the host’s own digestive limitations [61,62].

In summary, this study comprehensively revealed the gut microbiota of herbivorous grass carp, omnivorous carp, and crucian carp, which are closely related to diet and environment in structure and function. From nutrition acquisition and immune regulation to health maintenance, gut microbes play a key and unique role in the life activities of fish with different feeding habits. Feeding habits, as the dominant factor, shape the community structure and functional characteristics of intestinal microorganisms, and intestinal microorganisms react to the host through various metabolic pathways and immune regulation mechanisms to help fish adapt to their own living environment. The results of this study provide a detailed basis for further understanding the symbiotic mechanism between freshwater fish and intestinal microorganisms and also provide theoretical support and practical guidance for maintaining the intestinal microecological balance of fish and improving the health and breeding efficiency of fish by optimizing feed formula and regulating breeding environment in aquaculture. Aquaculture plays an important role in economic development. Through the effective use of saline–alkali water for aquaculture, we can optimize the utilization of water resources and improve the economic benefits and long-term growth of saline–alkali water fisheries.

## 5. Conclusions

In this study, the natural saline–alkali lake in Xinjiang in winter was taken as the unique research object. The 16S rRNA sequencing technology was used to systematically analyze the microbial community structure of the intestine and water of herbivorous (*grass carp*) and omnivorous (*carp*, *crucian carp*) fish in this extreme environment for the first time. The study confirmed, for the first time, that the gut bacterial communities of herbivorous and omnivorous fish formed significantly different clusters under high saline–alkaline and low temperature stress in winter in Xinjiang, and the composition of gut microbiota in both types of fish was significantly different from that in water (*p* < 0.05). At the same time, the gut microbial communities of different fish species were also significantly different. It was further found that the low temperature environment significantly inhibited the growth of thermophilic microorganisms, and the low temperature adaptive bacteria, such as *Shewanella* and *Psychromonas*, became the dominant colonization flora of grass carp intestine, which was the unique environmental adaptation characteristic of fish intestinal flora in the saline–alkali lake in winter and provided a new perspective for the study of fish intestinal flora interaction in extreme environments. This study only covers a single period in winter and lacks seasonal dynamic data. In the future, it is necessary to carry out cross-seasonal monitoring, combine multi-omics to verify the function of flora, and verify the effect of probiotics through breeding experiments so as to promote the transformation of basic research into the industry.

## Figures and Tables

**Table 1:** Physical and chemical indicators of water at sampling points (S1–S5).

	S1	S2	S3	S4	S5
Temperature (°C)	0.5	0.3	0.65	0.4	0.52
DO (mg/L)	9.56	9.34	9.15	8.12	9.68
PH	8.54	7.55	8.32	8.25	8.23
Salinity (ppt)	6.52	6.21	6.14	6.77	6.35
TDS (mg/L)	7428.65	7139.10	7109.36	6946.15	6953.14

**Table 2:** Alpha diversity index of different samples. GC: grass carp; CC: carp; GRC: crucian carp; WT: water samples.

	GC	CC	GRC	WT
Chao1	478.1596 ± 217.7226 c	2160.6622 ± 494.0034 a	2159.0822 ± 310.1022 a	1551.2975 ± 77.5678 b
ACE	490.0839 ± 223.0027 c	2197.9752 ± 501.3696 a	2206.0507 ± 314.0983 a	1570.3253 ± 78.4579 b
Simpson	0.9189 ± 0.0487 b	0.9558 ± 0.032 ab	0.9309 ± 0.0427 ab	0.9706 ± 0.0025 a
Shannon	5.313 ± 0.7933 b	7.2329 ± 1.1022 a	6.9461 ± 0.7936 a	6.9377 ± 0.1421 a
Coverage	0.9989 ± 0.0006	0.9957 ± 0.0008	0.9951 ± 0.0009	0.9986 ± 0.0003

Note: Different letters in the same row indicate significant differences (*p* < 0.05).

## Data Availability

The original contributions presented in this study are included in the article. Further inquiries can be directed to the corresponding authors.
